# Evaluation of psychometric properties of perceived value applied to universities

**DOI:** 10.1371/journal.pone.0284351

**Published:** 2023-04-11

**Authors:** Marelby Amado-Mateus, Yonni Angel Cuero-Acosta, Alfredo Guzman-Rincón

**Affiliations:** 1 Business School, Universidad del Rosario, Bogotá, Colombia; 2 School of Economic and Administrative Sciences, Corporación Universitaria de Asturias, Bogotá, Colombia; St John’s University, UNITED STATES

## Abstract

Over the past 20 years, the construct of perceived value has been the subject of much research, most of it applied to the service sector. The intangible nature of this sector requires an in-depth analysis of customer perceptions of what they give and what they receive. In this research, perceived value is applied in the context of higher education, where perceived quality faces several challenges and has a tangible component that is related to their experience when receiving the educational service, and an intangible component that is related to the image and reputation of the university. One of these challenges is the increasingly competitive environment of universities, so it is important to understand what factors influence students’ perception of value. For this purpose, several scales of perceived value were reviewed and one was selected and its psychometric properties were evaluated. For this evaluation, cultural adaptation techniques, exploratory factor analysis and confirmatory factor analysis were used. The statistical results showed the validity and reliability of the scale applied to universities in the Colombian context.

## 1. Introduction

The higher education sector has been impacted by the globalisation changes, as well as the high competition at local, national, and international levels [1]. At the same time, there is a growing educational trends with an important virtual component which is taking over of most part of the demanding service. These factors have forced universities to change their social and educational focus to a more commercial, market-oriented approach [2,3]. Consequently, higher education institutions (hereafter HEIs) administrators have had to manage their internal and external processes to attract students, position the institution with marketing differentials, reduce ineffective recruitment, and mitigate poor data management as well as inadequate student care [4]. This means that HEIs prioritise the attraction of students in parallel of focusing on improving the quality of the provided education service as these factors are related to a better perception of value by students [5].

In an attempt to improve quality, HEIs have opted for high assurance quality certification [6] at the local level; for example, in the Colombian case with the national governmental high assurance quality accreditation system for higher education institutions. At the international level, for example, this happens through the main accreditation agencies such as the European Quality Assurance Agency [EQAA] and the Accreditation Service for International Schools, Colleges and Universities [ASIC]. These certifications become criteria that students value positively when it comes to selecting the university where they will study [6].

Furthermore, the HEIs choose to enhance the student’s educational experiences and thereby generate better perceptions of value [7]. Managing student perceptions involves considering the quality of the education service from an objective approach where physical, technical, and technological aspects are addressed. From the subjective approach involving other components [8] as well as the reputation and image of the university. However, such management from the concept of perceived value (PV) presents ambiguity, as each student has his or her own perception of what quality and value are for them [9].

Other authors observe that quality management in higher education focuses on two dimensions, one of the service where it refers to the tangible (facilities, cafeterias, libraries, etc.) and a second, which is the educational dimension focused on teaching, research, and community relations [10]. Although quality management in HEIs in Colombia is quite similar, this research is limited to the tangible dimension. In this sense, marketing and communications play a relevant role in knowing and confirming students’ expectations about the education service that they will receive and its impact on their professional profile in order to be employed in an organisation or to generate their own company.

Authors like Matarranz and García-Madariaga [6] address the mercantilist approach that has generated a debate between whether or not to recognise the student as a customer [11] as some authors indicate that this approach blurs educational standards and undermines the teacher-student relationship [12]. It also creates a short-term vision, and sacrifices educational and social goals that are only recognised in the long term [13]. More recent research has concluded that digital marketing has a significant impact on student relationship management [14], but that students can be considered as customers, only in the recruitment process but not in the educational one [6]. Thus, the student is understood as "a collaborative partner" [13] where his active role in the educational service is recognised, therefore the PV value is the result of a "collaborative act of consumption" [15].

This collaborative act of consumption implies that university managers, faculty and support staff develop a management focused on the value perceived by the student, as this can increase the probability of success of organisations [16]. It can also have an impact on improving university performance as well as on profitability [1]. However, understanding the concept of PV is a complex task not only because of the characteristics attached to the service, such as intangibility, separability, and expiration, among others [8]. But also rendered complex by the particularities of higher education, such as student participation in their training process, the satisfaction of needs through the various stakeholders, continuity of service and the permanence of services that do not expire in a short period of time [5]. In view of the difficulty of conceptualising PV [17], there are also difficulties in its valuation, nevertheless, empirical studies have addressed the measurement of value through quality from the perspective of students using and adapting, as mentioned by Wong and Sultan [18], instruments such as the SERVQUAL, the SERVPERF and the HEdPERF.

It is worth noting, various proposals by authors who have sought to evaluate the PV of universities were reviewed, such as Ledden et al. [19] and Clemes et al., [20], among others. They have sought to understand students’ perceptions and relationships with other constructs to improve the student’s academic journey, in line with the rigorousness of the formative and experiential processes that directly influence the student’s teaching-learning process [21]. From this perspective, this article makes a cultural adaptation of the VP scale developed by Ledden et al. [19] to the Colombian context. In this regard, according to Gjersing et al. [22] there is no universal agreement on how to adapt the instrument in different cultural environments, however, these adaptations are very useful to precisely describe what is being measured, avoiding biases and enabling comparison between the results found in different countries.

Similarly, it is important to recognize that in studies of economic and administrative sciences it is unusual to publish studies on the validation and cultural adaptation of scales, since the process is only tangentially mentioned. For this reason, the present research takes as a reference the process used by Hosseini et al. [23] to evaluate the psychometric properties of learning-teaching experiences of nursing students. The process proposed by the authors includes translation and back-translation, participant selection, content validity and the response process.

In addition to the process of validation and adaptation of the scale, this research is expected to evaluate the ability of the scale to measure the construct of perceived value. This study provides a first approach to the validation of the scale from the *Covariance-Based Structural Equation Modeling* (CB-SEM) approach. To this end, this article first presents a conceptual approach to perceived value; secondly, it describes the methodology and process used; thirdly, it shows the results obtained; and finally, it presents a discussion of the findings and conclusions.

## 2. Theoretical framework

### 2.1 Concept of perceived value

The concept of value is one of the foundations of marketing [24] and its development took place shortly before the 1990s [25]. In this respect, one of the first and most relevant definitions is that proposed by Zeithaml [26], who defined value as the consumer’s evaluation of a product or service, based on his perception of the result of the exchange between what he gives and what he receives. From there, other contributions have been generated around the same idea [27,28]. According to consumer value theory, PV attempts to explain why consumers buy what they buy, suggesting five main categories of consumer values [29,30]:

1. Functional value (related attributes, physical, and utilitarian performance)
2. social value (symbolic and group belonging)
3. emotional value (affective responses)
4. epistemic value (curiosity, innovation, and knowledge)
5. conditional value (specific situation)

Despite its development in the area of marketing, the concept of value has interpretations such as value, utility, price and quality [31], and even as a quality of people. Similarly, value is understood as the result of an evaluative judgement, while the term values refer to the standards, rules, criteria, objectives, or ideals that serve as the basis for such an evaluative judgement [32]. Value then is a judgement of preference and values are the criteria by which people make such judgements, in short, the two concepts are related, but at the same time, they are distinct [19].

The functional concept of value is developed in the purchase or use of a product or service and is evidenced by the recognition of the benefit obtained and the sacrifice made by the consumer based on his or her expectations [27]. Consequently, value is more focused on the evaluation of the perceived performance of a product or even a service, in terms of its attributes and characteristics such as ease of use and disposal, considered preferable for the achievement of its particular purposes and objectives [28]. It should be noted that value is a different concept from quality, although they are related, as quality is an antecedent of value [33,34].

In the same sense, PV is the result of evaluating the usefulness of the product or service through the perception of the benefit obtained versus the cost of that product [19,35]. In addition, the alternatives available and their comparative nature are considered, specifying that there is a PV between the different offers or alternatives offered by the suppliers [25]. Likewise, it is understood as a cognitive-affective evaluation of the exchange relationships within any of the stages of the purchase decision process, which includes tangible and intangible elements that play a transcendent role in the decisions and in the comparison made by the customer, where factors such as time, place, and the circumstances of evaluation, among others, converge [31].

PV is the result of marketing actions; its importance lies in the fact that it influences the customer’s decision making, which in turn influences their intention to repurchase and the intention to share with others the positive messages through word of mouth and the willingness to pay the price of the product or service, involving other factors such as perceived cost, sacrifice, intrinsic and extrinsic attributes [1]. In short, PV is a trade-off between the most important components of what the customer feels they give and the performance they receive, with an important influence on loyalty [36].

In the case of higher education, PV has been analysed by means of scales such as that of Clemes et al. [20] which sought to analyse the relationships between Chinese students’ behavioural intentions, satisfaction, service quality, PV, and university image and which was carried out considering previous scales [37], but adjusted by means of focus group carried out with students. The results showed a multilevel model composed of three main dimensions and 13 sub-dimensions to conceptualise and measure perceived service quality [20].

Another important research was the one conducted by Doña-Toledo et al. [1] which set out to validate a model of antecedents and consequences of the PV of university among graduates, and to analyse the moderating role of their level of engagement with higher education. The scale developed by Doña-Toledo et al. [1] to measure PV is an adaptation of the scale proposed by Zeithaml [26] and Cronin et al. [34], their results showed that perceived quality determines PV and this, in turn, decisively influences contentment. The scales also concluded that value determines satisfaction exclusively among graduates with a low level of involvement. The Ledden et al. [19] scale was selected for this research with the purpose to examine the functional relationship between personal values and PV in the educational sphere. This scale is selected because of its relevance and wide application in various countries in the education sector.

## 3. Methodology

### 3.1 Ethics statement

The research protocol was approved by the Research Ethics Committee of the Universidad del Rosario in the Social Sciences Room with an approval date of September 22, 2021, for human studies. Written informed consent was collected from each of the study participants.

### 3.2 Instrument

To measure the construct of PV, the instrument used is the one developed by Ledden et al. [19], who divide the concept of value into two dimensions and eight factors. The first dimension is "what you get", where six factors are assessed, namely functional value, epistemic value, social value, emotional value, conditional value, and image. In the second dimension of "what is given", two factors are assessed, monetary and non-monetary sacrifice. The instrument contains 26 items, and it uses a Likert scale from 1 to 7. The permission to use the instrument was requested to the corresponding author who, in addition, provided the original instrument, which was applied to his study.

### 3.3 Adaptation and data collection procedures

**Fig 1:**
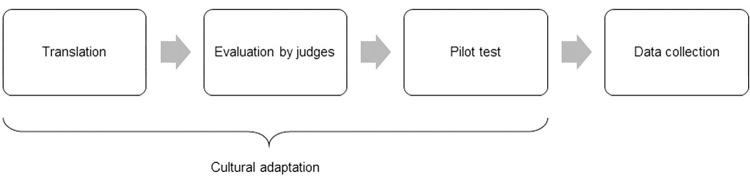
Adaptation procedures.

For the analysis of the PV scale, a four-step process of cultural adaptation and data collection was carried out (Adapted from Hosseini et al., [23]), as illustrated in Fig 1.

### 3.4. Translation

In order to adapt the scale, the first step was to translate it from English into Spanish. This process was carried out by two bilingual experts. Then, a reverse translation into English was made and sent to two experts with doctoral degrees, who reviewed the scales translated into English and presented their acceptance. Table 1 presents the codes and translations of the questions in both English and Spanish.

**Table 1:** Items in English and Spanish.

Factors	Code	English Items	Spanish Items
Functional value	TYTUC1	My degree will allow me to earn a good/better salary	Mi título universitario me permitirá ganar un buen / mejor salario
	TYTUC2	My degree will allow me to achieve my career goals	Mi título me permitirá alcanzar mis objetivos profesionales
	TYTUC4	My degree will lead to promotion in my current/future job	Mi título conducirá a un ascenso en mi trabajo actual o futuro
Epistemic value	ECDC1	The content of my course keeps me interested	El contenido de mi carrera me mantiene interesado
	ECDC2	I learn new things from my course	Aprendo cosas nuevas de mi carrera
	ECDC3	The course content contributes to the high value of my education	El contenido de mi carrera agrega valor a mi educación
	ECDC4	The academic guidance I receive from my lecturers has enhanced the value of my degree	La orientación académica que recibo de mis profesores agrega valor a mi título
Social value	TCROP1	People who are important to me think that taking my course is a good thing to do	Las personas que son importantes para mí piensan que cursar mi carrera es algo bueno
	TCROP2	People who influence what I do think that taking my course is a good idea	Las personas que influyen en mí creen que es una buena idea tomar mi carrera
	TCROP3	My current/future employer will see me in a better light when I have finished my degree	Mi empleador actual o futuro me verá mejor cuando termine mi carrera
	TCROP6	The support of my friends and family has been important in helping me through my course	El apoyo de mis amigos y familiares ha sido importante para ayudarme en mi carrera
Emocional value	TCPS1	I feel proud that I’m taking my course	Me siento orgulloso de estar cursando mi carrera
	TCPS2	Taking my course has boosted my self confidence	Cursar mi carrera ha aumentado mi autoestima
	TCPS5	Taking my course has given me a sense of self‐achievement	Cursar mi carrera me ha dado una sensación de autorrealización
Conditional value	OFRCC1	The support materials supplied to me on my course (e.g., study packs/texts) have helped my learning	Los materiales de apoyo que me proporcionaron en mi carrera (por ejemplo, libros/ textos de módulos) me han ayudado a aprender
	OFRCC2	Study‐group work has been a beneficial part of my course	El trabajo en grupo ha sido una parte beneficiosa de mi carrera
	OFRCC3	The Kingston Hill campus and its facilities have contributed to the value of my course	El campus y sus instalaciones han contribuido al valor de mi carrera
Non-monetary sacrifices	LSHSC2	My studies have reduced the time I spend with my family	Mis estudios han reducido el tiempo que paso con mi familia
	LSHSC3	My studies have reduced the time I spend with my friends	Mis estudios han reducido el tiempo que paso con mis amigos
Monetary sacrifices	LSHSC5	The monetary price paid for my course is reasonable when I consider what I am getting out of it	El precio monetario pagado por mi carrera es razonable cuando considero lo que obtengo de él
	LSHSC6	When considering the monetary price of my course, I believe that the quality is good	Considero que el precio monetario de mi carrera está acorde con la calidad
Image	LPAU1	The reputation of KBS influences the value of my degree	La reputación de mi universidad influye en el valor de mi título
	LPAU2	The image projected by KBS has an influence on the value of my degree	La imagen proyectada por mi universidad influye en el valor de mi título
	LPAU3	I believe that employers would have positive things to say about KB	Creo que los empleadores tendrían cosas positivas que decir sobre mi universidad
	LPAU4	I have heard positive things about KBS	He escuchado cosas positivas sobre mi universidad
	LPAU5	I believe that KBS has a good reputation	Creo que mi universidad tiene una buena reputación

### 3.5 Evaluation by judges

This evaluation was carried out by eight expert judges in marketing or management. To measure content validity and Aiken’s V statistic (V) was used to analyse the responses. The possible outcomes ranged from zero to one, where a value of one represents a perfect degree of agreement among the judges and a value of zero is general disagreement. The value of this statistic was considered acceptable at values equal to or greater than 0.7 [38]. For this study, the following aspects were assessed: relevance, pertinence, response induction, sufficiency, clarity and wording, as well as the scale of the response.

The content analysis of the present scale showed that for the aspects of relevance (V = 1.00), relevance (V = 1.00), response induction (V = 1.00), sufficiency (V = 0.86), as well as for clarity and wording (V = 0.92) the judges had a high level of agreement; only for the case of scale of the response it was considered acceptable, being the V = 0.71.

### 3.6 Pilot test

A pilot test was carried out to confirm the comprehensibility of the scale in undergraduate students at a private university in the field of management. This evaluation was carried out with a sample of twenty students who, in addition to answering the survey, provided feedback on the clarity and wording of both the scale and the initial prompts. The results of the assessment showed a clear understanding of the items and instructions, and no additional adjustments were necessary.

### 3.7 Participants

The scale was administered to students enrolled in undergraduate programmes and of legal age at two universities in the city of Bogotá, Colombia. A total of 484 surveys were collected. The sample consisted of 285 women and 199 men. Most of the students are between 18 and 21 years old (68%) and are in their second and third year of their studies. The highest share of responses was obtained from the following programmes: business administration, international business administration, logistics and production management, public accounting, market management technology, marketing and digital business, and psychology.

### 3.8 Statistical analysis

For the analysis of the data obtained, an Exploratory Factor Analysis (EFA) was developed and the preliminary findings of the exploratory one was verified by means of a Confirmatory Factor Analysis (CFA), which corresponds to the CB-SEM approach.

#### EFA

Based on the data collected, an EFA was conducted to determine the possible clustering of scale items among the sample of students. The statistic Kaiser-Meyer-Olkin (KMO) and the Bartlett’s test of sphericity (BTS) were used to check whether the data were suitable for this type of analysis. The principal factors method with varimax rotation was also used, eliminating items with factor loadings below 0.30 and taking as a reference the criteria established by Cronbach [39], Godfrey et al. [40] and Comrey and Lee [41].

#### CFA

For the CFA, the factors of the EFA were used as a basis for conformation of the factors. Univariate and multivariate normality was established for the items of each of the scales. For the operationalisation of the CFA, maximum likelihood estimates were made using the maximum likelihood. The bootstrap (i.e., 2,000 bootstrap samples with 95% confidence intervals) was also used with reference to Oppong and Agbedra [42] for this procedure. The following were taken into account for the CFA evaluation statistic: minimum discrepancy ratio (*X*^2^/*df*), comparative form index (*CFI*), normed fit index (*NFI*), Tucker-Lewis index (*TLI*), incremented fit index (*IFI*), goodness-of-fit- index (*GFI*), adjusted goodness-of-fit index (*AGFI*) and root mean square error of approximation (*RMSEA*). Where thresholds for *X*^2^/*df*<5, *CFI*, *NFI*, *TLI e IFI*>0.90, *GFI y AGFI*>0.80, and RMSEA<0.08. In Table 2, the description of each of the statistics and units of measurement is presented.

**Table 2:** Description of statistical test.

Statistical test	Description	Interval
*X*^2^/*df*	Determines the degree to which the model predicts the correlation matrix.	Not apply
*CFI*	It is a standardised fit index that compares the fit of a hypothetical model with that of a reference model.	0–1
*NFI*	Compares the proposed model and the null model considering an acceptable value	0–1
*TLI*	It allows evaluating a relative reduction of the mismatch per degree of freedom.	0–1
*IFI*	Adjusts the normalised fit index to the sample size and degrees of freedom of the selected model. of the selected model.	0–1
*GFI*	Assesses whether the model needs to be adjusted.	0–1
*AGFI*	It is an extension of the GFI, which adjusts the degrees of freedom between the reference and null models.	0–1
*RMSEA*	Is a measure of poor fit, i.e. an absolute fit index that allows one to assess how far a hypothetical model is from a perfect model.	0–1

Source: Useche et al. [43] and Escobedo et al. [44].

Following this, the convergent analysis of the factors of each of the scales was carried out, for which the Extracted Variance (AVE), reliability evaluated from the composite reliability (CR) statistic, as well as Cronbach’s Alpha (α) were determined. For AVE, values greater than 0.5 were considered acceptable, for CR values greater than 0.7, and for α values greater than 0.8. Finally, for the divergent analysis of the factors, the Maximum Shared Variance (MSV) and Average Shared Variance (ASV) statistics were calculated, being considered acceptable when MSV and ASV are lower than AVE.

## 4. Results

The statistical analyses showed that the KMO was 0.96 indicating the partial correlations of the variables assessed by the scales, and the Barrllett’s Test of Sphericity gave a value of *X*^2^ = 23,223.96 with p-value < 0.001, giving a good fit for the application of the EFA. With the conformation of the seven factors, 80.83% of the explained variance was explained. Table 3 shows the factor loadings.

**Table 3:** Factor loadings on the EFA perceived value scale.

Variable	1	2	3	4	5	6	7
TYTUC1	0.67						
TYTUC2	0.57						
TYTUC4	0.60						
ECDC1		0.74					
ECDC2		0.77					
ECDC3		0.76					
ECDC4		0.69					
TCROP1			0.79				
TCROP2			0.80				
TCROP3			0.30				
TCROP6			0.60				
TCPS1				0.53			
TCPS2				0.68			
TCPS5				0.68			
OFRCC1		0.60					
OFRCC2							0.31
OFRCC3							0.32
LSHSC2					0.98		
LSHSC3					0.86		
LSHSC5						0.78	
LSHSC6						0.79	
LPAU1							0.84
LPAU2							0.85
LPAU3							0.65
LPAU4							0.59
LPAU5							0.44

**Fig 2:**
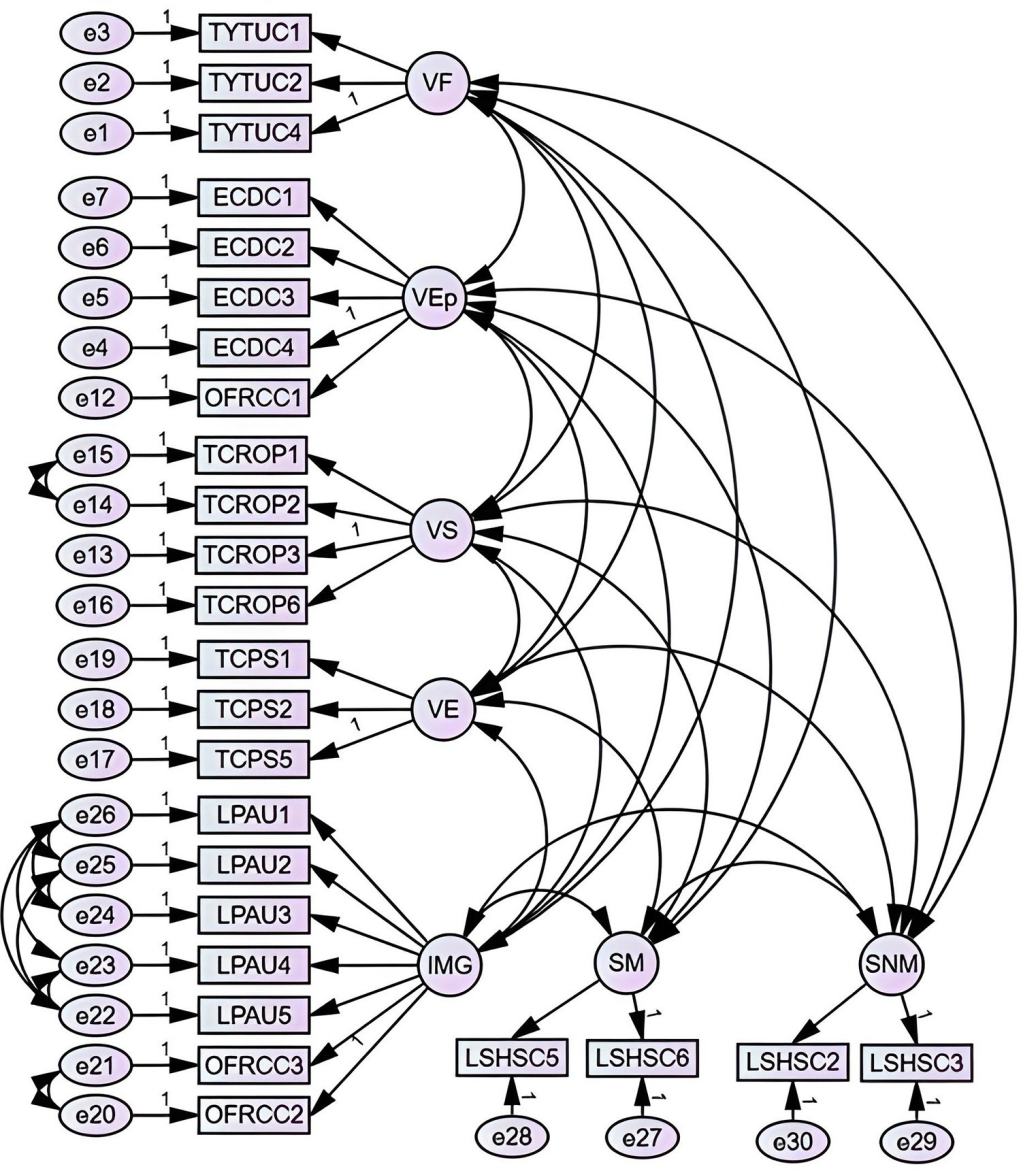
CFA with error covariation.

Based on the factor analysis, the development of the CFA was proceeded. The results of the model’s fit statistics were as follows CFA *χ*^2^/*df* = 6.03, *CFI* = 0.90 *NFI* = 0.83, *TLI* = 0.90, *IFI* = 0.90, *GFI* = 0.77, *AGFI* = 0.72 and *RMSEA* = 0.01. Based on the above, no standardised regression weights of less than 0.60 were detected, so no variables were eliminated, and the errors were covaried according to the most parsimonious modification indices as shown in Fig 2.

With the modifications developed in the standard CFA the new values of the statistics were *χ*^2^/*df* = 3.81, *CFI* = 0.94 *NFI* = 0.92, *TLI* = 0.93, *IFI* = 0.94, *GFI* = 0.85, *AGFI* = 0.81 and *RMSEA* = 0.07, which represented a good fit. Table 4 presents the weights of the standardised loadings for each of the variables. On the other hand, Table 5 shows the weights of the standardised loadings result of bootstrap samples.

**Table 4:** Standardised regression weights for perceived value scale.

Factor	Variable	1	2	3	4	5	6	7
Functional value	TYTUC1	0.91						
	TYTUC2	0.94						
	TYTUC4	0.90						
Epistemic value	ECDC1		0.91					
	ECDC2		0.95					
	ECDC3		0.97					
	ECDC4		0.90					
	OFRCC1		0.78					
Social value	TCROP1			0.83				
	TCROP2			0.85				
	TCROP3			0.80				
	TCROP6			0.83				
Emotional value	TCPS1				0.91			
	TCPS2				0.86			
	TCPS5				0.90			
Non-monetary sacrifices	LSHSC2							0.91
	LSHSC3							0.93
Monetary sacrifices	LSHSC5						0.86	
	LSHSC6						0.96	
Image	OFRCC2					0.72		
	OFRCC3					0.65		
	LPAU1					0.67		
	LPAU2					0.72		
	LPAU3					0.85		
	LPAU4					0.88		
	LPAU5					0.90		

**Table 5:** Results of bootstrap samples.

Factor	Variable	Estimate	Confidence intervals	P-Value
Functional value	TYTUC1	0.90	[0.86, 0.93]	0.001
	TYTUC2	0.94	[0.91, 0.9]	0.001
	TYTUC4	0.91	[0.88, 0.93]	0.001
Epistemic value	ECDC1	0.90	[0.86, 0.93]	0.001
	ECDC2	0.96	[0.95, 0.98]	0.001
	ECDC3	0.95	[0.92, 0.96]	0.001
	ECDC4	0.91	[0.88, 0.93]	0.001
	OFRCC1	0.78	[0.71, 0.83]	0.001
Social value	TCROP1	0.80	[0.70, 0.86]	0.001
	TCROP2	0.85	[0.75, 0.91]	0.001
	TCROP3	0.82	[0.74, 0.88]	0.001
	TCROP6	0.83	[0.73, 0.89]	0.001
Emotional value	TCPS1	0.90	[0.84, 0.94]	0.001
	TCPS2	0.85	[0.79, 0.90]	0.001
	TCPS5	0.90	[0.85, 0.94]	0.001
Non-monetary sacrifices	LSHSC2	0.63	[0.54, 0.71]	0.001
	LSHSC3	0.71	[0.63, 0.77]	0.001
Monetary sacrifices	LSHSC5	0.88	[0.83, 0.92]	0.001
	LSHSC6	0.87	[0.81, 0.91]	0.001
Image	OFRCC2	0.88	[0.824, 0.93]	0.001
	OFRCC3	0.78	[0.688, 0.85]	0.001
	LPAU1	0.74	[0.656, 0.82]	0.001
	LPAU2	0.96	[0.924, 0.99]	0.001
	LPAU3	0.85	[0.805, 0.90]	0.001
	LPAU4	0.92	[0.838, 102]	0.001
	LPAU5	0.90	[0.809, 100]	0.001

Based on the weights of the standardised regressions, it is identified that the scale has adequate convergent validity for all the factors, showing that, for the factors of functional value, epistemic value, emotional value and non-monetary sacrifice, the value of the AVE was higher using the CFA than the AVE developed by the PLS-SEM method of the original scale. Regarding discriminant validity, it was observed that there are indications that, for the social value and image factors, there are high correlations with the other factors; however, this is not conclusive given that for these factors the ASV is lower than the AVE. Table 6 shows the values of the convergent and discriminant validity statistics.

**Table 6:** Convergent and discriminant validation statistics perceived value scale.

Factor	Variable	α	AVE	CR	MSV	ASV	AVE^a^
Functional value	TYTUC1	0.94	0.84	0.94	0.74	0.52	0.71
	TYTUC2						
	TYTUC4						
Epistemic value	ECDC1	0.95	0.81	0.95	0.74	0.54	0.67
	ECDC2						
	ECDC3						
	ECDC4						
	OFRCC1						
Social value	TCROP1	0.90	0.65	0.89	0.83	0.55	0.66
	TCROP2						
	TCROP3						
	TCROP6						
Emotional value	TCPS1	0.91	0.79	0.92	0.67	0.49	0.64
	TCPS2						
	TCPS5						
Non-monetary sacrifices	LSHSC2	0.91	0.84	0.91	0.07	0.05	0.88
	LSHSC3						
Monetary sacrifices	LSHSC5	0.90	0.83	0.90	0.66	0.40	0.73
	LSHSC6						
Image	OFRCC2	0.92	0.70	0.91	0.83	0.53	0.72
	OFRCC3						
	LPAU1						
	LPAU2						
	LPAU3						
	LPAU4						
	LPAU5						

^a^ This AVE corresponds to the original instrument results and is placed for comparative purposes.

## 5. Discussion of the results

### 5.1 Regarding psychometric properties

The results obtained in the PV scale show that the items were grouped into seven factors (see Table 3), and not eight, as in the original scale. The factor that was disseminated was the "conditional value" factor, which consisted of three items related to other factors in the study programme. In the analyses performed, the items were like other values, so they did not have the capacity to assess the factor and were disintegrated. The items of this factor were as follows, OFRCC1 “The support materials supplied to me on my course (e.g., study packs/texts) have helped my learning”, OFRCC2 “Study‐group work has been a beneficial part of my course”, OFRCC3 “The campus and its facilities have contributed to the value of my course”. Within the epistemic value, which addressed the issue of career content, item OFRCC1, which relates to support materials, was included, as it was understood as a more career-related characteristic and not as another factor. As for the items OFRCC2 and OFRCC3, the first related to teamwork and the second related to facilities, were included in the "image" factor, and if the two items are reviewed in depth. OFRCC3 is more related to the emotional component of the image as expressed by Nguyen and Leblanc [45], since they are aspects resulting from the experiences that the students have with the organisation. The OFRCC3 item refers to one of the components related to the cognitive image that includes the knowledge and perceptions that the student has regarding the facilities, courses, requirements, among others [46].

Likewise, in the analysis of the results obtained in the study developed by Kubat [47], who also used the scale developed by Ledden et al. [19] it was necessary to eliminate one factor to achieve a better fit of the model, leaving the scale with seven factors. Also, this study required disseminating one of the factors of the model but the values, functional, epistemic, social, emotional, and image are still maintained in the dimension of receiving, while in the dimension of giving, monetary sacrifices and non-monetary sacrifices are still retained, with a total of 26 items, which were the original ones since it was not necessary to eliminate any of them.

### 5.2 Regarding the comparison with previous studies

For Ledden et al. [19] the value is conceptualised as a higher-order formative latent variable, i.e. an index of its constituent dimensions. Its scales are the product of modifications of other scales developed by different authors such as LeBlanc and Nguyen [48], Cronin et al. [33] and Haistead et al. [49]. To further elaborate on the construction of the scale proposed by Ledden, the original authors were reviewed. LeBlanc and Nguyen [48], proposed a 33-item scale that addressed different aspects of a business school’s service offering as components of PV, including functional value (which focused on want /satisfaction), epistemic value (knowledge), image (included image and reputation), emotional value (relationship with the course and the personal effort), functional value (which grouped aspect of price/quality) and social value. On the other hand, Cronin et al. [33] conducted a study relating the effects of service quality, satisfaction and value on consumers’ behavioural intentions in six service industries. Haistead et al. [49] proposed a model, in which student satisfaction with higher education is a function of two attributes, performance and disconfirmation: intellectual environment and job readiness, which was tested with business school graduates at Eastern University in the United States.

Subsequent studies tested the scales proposed by Ledden et al. [19] with the following results. Kubat [47] conducted a study to analyse the relationships between personal values, PV of education and satisfaction among students at Akdeniz University in Turkey. As mentioned above, this study took the scales of Ledden et al. [19] to measure PV, from the dimension of giving and receiving, and the satisfaction. Their results showed that PV is a predictor of satisfaction, and that the dimension of receiving is removed to relate the values directly, finding that social value had the greatest influence, and that image had the least influence, it was also identified that, for students, the reputation of the university is not of great value, in the resulting model the non-monetary sacrifice was also removed. The study conducted by Matarranz and García-Madariaga [6] to measure the relationship between perceived quality, PV and repurchase intention of former graduate students at a university in the United States, used part of the Ledden et al. [19] scale, taking from the giving dimension only the items related to functional value and some items from the receiving scale, as well as a modification of the scale by Dlačić et al. [50] scale to measure perceived quality independently, and their findings show a relationship between perceived quality and PV, and between PV and repurchase. Dlačić et al. [50] postulated PV as a multifaceted concept, divided into three, the functional aspects of the experience, the students’ emotions, and the comparison with other alternatives.

Following up on research developments regarding PV in postgraduate higher education programmes, Ledden et al. [15] adapt their model, keeping the dimensions of giving and receiving, but notably reducing the items, especially in the dimension of receiving, where the functional, epistemic and other values are found, in general in the dimension of what is given, the authors make modifications in the monetary and non-monetary sacrifices and use the approach of sacrifices of money and time, keeping 5 of its 6 items. A fundamental change in the model is that the satisfaction dimension is treated as a concrete attribute with only one measure. The adaptation made by the original authors of the scale shows that it is still important to further analyse the issue of value, adjusting it to different types of institutions (private—public) and students, whether undergraduate, postgraduate, or doctoral, since at certain stages of academic life, perceptions of value may be different

In the case of Latin America and especially Colombia, as Peña [51] states, the results given in the previous studies are not generalizable. However, the results obtained were very similar to those proposed by the authors, and the contributions of the study of PV in HEIs carried out in Colombia by Serna-Loaiza et al. [52] and applied to 1569 students in virtual mode, with a scale composed of 83 items, which resulted in the conformation of 10 factors that influenced the PV: functional value, emotional value, social value, epistemic value, conditional value, spiritual value, and ethical value and ethical-conditional value, socio-emotional value, ethical-functional value, ethical-social value, and ethical-spiritual value.

## 6. Conclusions

The quality of higher education in Ibero-America continues to be a challenge. According to the OEI [53] among the challenges faced by institutions, belonging to this sector is the increase of private universities with lower quality standards in their offerings and deficiencies in quality assurance systems, which has affected the competitive position of Ibero-American universities in international rankings such as the Academic Ranking of World Universities (ARWU).

This situation has led universities, especially private universities, to seek different alternatives that allow them to differentiate themselves and add value to their academic offerings in order to attract both students -with the aim of ensuring economic sustainability- and qualified teachers with a research profile that will allow them to improve their research indicators. However, in this context of inclusion of different strategies, it is not clear to managers how to measure the value perceived by their main interest group, which is the students. For this reason, the objective of this study was to analyse the psychometric properties of the Spanish adaptation of the PV scale in a population of university students.

For HEIs in Colombia, the validation of this scale will not only provide a tool for collecting information regarding perceptions of the value of the student experience. It is also a call for HEIs to focus their management and relationship efforts towards students in terms of creating value and ensuring results within the programs that the student decides to study. In this aspect, it is important to mention that the student not only perceives the quality of education from the contents of the learning process, but is also susceptible to those aspects in which he/she analyzes the value of the tuition and the good name that he/she can obtain upon graduating from a given higher education institution. Likewise, when selecting a HEI, students will always take into account the perception of the institution in terms of its reputation and image in the context of the country.

For HEI managers, it is important to link the management of student perception to the strategic plans of the institutions and to consolidate the strategic planning axes around the link between the quality of education and the elements that students value when acquiring this level of training. This will allow managers to focus their strategies and resources on the factors that generate value for the student, and with this, a sustainable differential that allows them to improve their academic program offerings and overcome those quality challenges that persist in the higher education system in Colombia.

Finally, with regard to the statistical results of the scale, it was found that confirmatory factor analysis for the Colombian case showed a model very close to the original one, with seven factors, evidencing that there was consistency between the theoretical factor structures [19] and the empirical factor structure. Despite the cultural differences of Colombian students, combined with the differences between the education systems compared between the UK and Colombia, the results are similar, showing the validity and reliability of the scale. In fact, as observed in Table 6, the results of the AVE of this study compared to the AVE of the original study are higher in most of the factors, such as functional, epistemic, emotional, and monetary sacrifices.

## 7. Limitations

The results provided evidence of a scale that measures, through the seven factors, the value that students perceive of their university experience and serves not only for empirical research purposes but also for practical purposes, so that university administrators can learn about their students’ perceptions and act with respect to the processes and procedures they develop to improve students’ experiences. However, the researchers recognise that further empirical research is needed to test the adaptability and measurability of the value construct in other cultural contexts, and even in the Colombian context.

Similarly, there are limitations regarding the sample, since students from undergraduate academic programs were taken into consideration. Therefore, it is recommended to include other profiles such as graduate students in future research. Likewise, although the scale was validated for a specific sample and with good statistical results, this does not guarantee that it will have the same results in other populations and with different objectives. It is also important to emphasize that the differences between the educational systems of Spanish-speaking countries may influence how perceived value is conceived, which is why a cross-cultural evaluation is recommended before using the scale.
